# Comorbid depression and obesity, and its transition on the risk of functional disability among middle-aged and older Chinese: a cohort study

**DOI:** 10.1186/s12877-022-02972-1

**Published:** 2022-04-03

**Authors:** Li Lin, Shigen Bai, Kang Qin, Carlos King Ho Wong, Tingting Wu, Dezhong Chen, Ciyong Lu, Weiqing Chen, Vivian Yawei Guo

**Affiliations:** 1 Department of Epidemiology, School of Public Health, Sun Yat-sen University, 74 Zhongshan Second Road, Guangzhou, 510080 Guangdong China; 2 Department of Family Medicine and Primary Care, LKS Faculty of Medicine, The University of Hong Kong, Hong Kong, SAR China; 3 Department of Pharmacology and Pharmacy, LKS Faculty of Medicine, The University of Hong Kong, Hong Kong, SAR China

**Keywords:** Depression, Obesity status, Comorbidity, Transition, Functional disability, Longitudinal study

## Abstract

**Background:**

Evidence has indicated that depression and obesity were associated with functional disability, independently. However, little is known about the detrimental impact of comorbid depression and obesity, as well as its transition on functional disability. This study investigated the association of baseline depression-obesity status and its dynamic change with incident functional disability among middle-aged and older Chinese.

**Methods:**

This cohort study included 5507 participants aged ≥45 years from the 2011 and 2015 waves of China Health and Retirement Longitudinal Study. Depression was defined with a score ≥ 10 using the 10-item Centre for Epidemiologic Studies Depression Scale. Obesity was defined as body mass index ≥28 kg/m^2^. Participants were cross-classified by depression and obesity status at baseline, and its change during follow-up. Logistic regression models were constructed to evaluate the association of baseline depression-obesity status and its transition with incident functional disability defined by the Katz index of activities of daily living scale.

**Results:**

Over four-year follow-up, 510 (9.3%) participants developed functional disability. Individuals with baseline comorbid depression and obesity had the highest risk of functional disability (OR = 2.84, 95% CI: 1.95–4.15) than non-depressive participants without obesity, or those with depression or obesity alone. When investigating the dynamic changes of depression-obesity status on functional disability incidence, those with stable comorbidity throughout two surveys had the greatest risk of functional disability (OR = 4.06, 95% CI: 2.11–7.80). Progression of depression-obesity status was associated with increased risk of functional disability, while regression from baseline to follow-up was linked to attenuated risk estimates.

**Conclusions:**

Among middle-aged and older Chinese adults, the risk of functional disability was exaggerated with comorbid depression and obesity. Our data further suggest that transitions of depression and obesity over time are associated with the risk of developing functional disability.

**Supplementary Information:**

The online version contains supplementary material available at 10.1186/s12877-022-02972-1.

## Background

With ageing population growing worldwide, most countries have to face the increasing burden of ageing-related health issues. One of such concerns is functional disability, which refers to the impairment of the capacity that enable people to be and to do what they have reason to value [1]. A cross-sectional study from 23 countries has shown that the average prevalence of functional disability measured by activities of daily living (ADLs) in women and men aged between 55 and 65 years was 7.9 and 6.9%, respectively [2]. In the context of China, the country with the largest ageing population in the world, the figures were even higher, with 19.8% in women and 12.1% in men at similar age [2]. Individuals with functional disability were associated with poorer physical health [3], reduced quality of life [4], and higher risk of mortality [5]. Therefore, great efforts have been made to identify the risk factors and high-risk groups of functional disability.

Depression and obesity are common in older people nowadays. Both conditions have been found to be associated with increased risk of functional disability [6–8]. Additionally, these two conditions often occur simultaneously with a bidirectional association due to shared genetic, metabolic, and psychosocial pathways [9, 10]. A meta-analysis of 19 studies has demonstrated that depressive people had 37% higher risk of having obesity than non-depressive individuals, while those being obese were 18% more likely to have depression than their non-obese counterparts [11]. Such reciprocal associations might exaggerate the detrimental impact on health caused by depression or obesity alone. Previous studies have therefore demonstrated that comorbid depression and obesity have been linked to greater risk of diabetes mellitus, relative to individuals with either condition alone [12, 13]. A recent study in China has further revealed that individuals with both depression and obesity had the greatest risk of prevalent functional disability compared to those with neither condition, or with either condition alone [14]. However, this study was based on cross-sectional data, which cannot assess the temporal association of comorbid depression and obesity with the risk of functional disability [14]. Additionally, it remains unclear how depression-obesity status changes over time and how such dynamic change affects the risk of functional disability among Chinese adults. This is an important issue with both public health and clinical significance, as the findings might inform prevention and intervention strategies for functional disability in ageing populations.

Therefore, this cohort study aimed to investigate (1) the association between baseline depression-obesity status and the risk of incident functional disability among middle-aged and older adults in China, and (2) whether transition of depression-obesity status over time can affect the risk of incident functional disability. Our findings may provide insight about whether depressive symptom should also be screened in addition to body mass index (BMI) among middle-aged and older adults in clinical practice. Furthermore, the results can help identify high-risk individuals of developing functional disability who may benefit from health intervention.

## Methods

### Study design and population

**Fig. 1:**
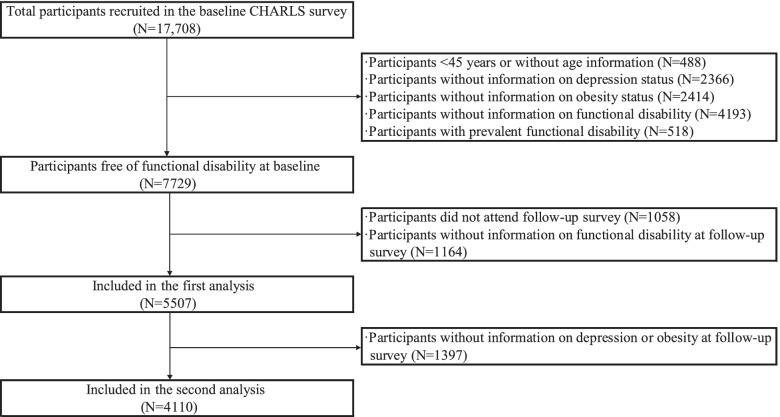
Flow chart of participants selection

Participants of this cohort study were recruited from the China Health and Retirement Longitudinal Study (CHARLS), a nationally representative survey of Chinese aged 45 years and older [15–17]. The baseline survey was conducted between 2011 and 2012 in 450 villages/resident communities of 28 provinces across China through multistage probability-proportional-to-size (PPS) sampling method. Of 17,708 participants recruited at baseline, we excluded 488 participants without age information or aged under 45 years, 2366 without information on depression, 2414 without information on obesity status, 4193 without assessment of functional disability, and 518 with prevalent functional disability, leaving 7729 participants free of functional disability from the baseline survey (shown in Fig. 1). Then, we excluded 1058 individuals lost to follow-up and 1164 individuals without assessment of functional disability in the 2015 survey. Finally, a total of 5507 eligible participants were included in the first analysis of the association between baseline depression-obesity status and incident functional disability. Regarding the transition of depression-obesity status over time, we further excluded 1397 participants without information on depression or obesity at follow-up, leaving 4110 individuals in the second analysis.

The ethics of CHARLS study was approved by the Institutional Review Board (IRB) at Peking University (IRB approval number: IRB00001052–11015 and IRB00001052–11014.14) [15]. Each participant has signed a written consent form. All methods were performed in accordance with the relevant guidelines and regulations.

### Assessment of functional disability

Functional disability was evaluated using the Katz index of independence in ADLs, which includes six items of dressing, bathing, eating, transferring, toileting, and continence [18]. Each item has four possible choices: ‘No, I don’t have any difficulty’, ‘I have difficulty but can still do it’, ‘Yes, I have difficulty and need help’, and ‘I cannot do it’. The participant was considered as having functional disability if he or she needed help or could not do any of the six activities, which was in line with previous studies [19]. The Katz index has been widely used in epidemiology practices and demonstrated to be reliable and valid among older adults [20].

### Measures of depression and obesity

Depression was evaluated over the previous 7 days using the Chinese version of the 10-item Centre for Epidemiological Studies Depression Scale (CESD-10) [21], which has been demonstrated to be reliable and valid to detect depression in Chinese adults [22, 23]. The total scores of CESD-10 ranged from 0 to 30, with higher scores reflecting more severe depressive symptoms. Depression was defined with a cut-off score of 10 [21].

The standing height and weight were measured by a standardized stadiometer (Seca™213, Seca Co., Ltd., Hangzhou, China) and a validated scale (Omron™HN-286 scale, Krill Technology Co., Ltd., Yangzhou, China), respectively. BMI was calculated as weight (kg) divided by the square of height (m^2^). According to the recommendations of Working Group on Obesity in China, obesity was defined as BMI ≥ 28 kg/m^2^ in Chinese adults [24].

All participants were divided into four groups according to the baseline depression and obesity status: neither depression nor obesity (neither condition); depression without obesity (depression alone); obesity without depression (obesity alone); with both depression and obesity (comorbidity).

### Covariates

Face-to-face interviews were used to collect information of socio-demographic and lifestyle factors, including age, gender, education level (illiterate or no formal education, primary school, and middle school or above), current marital status (married or cohabitated, and not married), area of residence (urban and rural), smoking status (never and ever smoker), and drinking status (never and ever drinker).

Systolic blood pressure (BP) and diastolic BP were measured three times with at least 45-s intervals by an electronic sphygmomanometer (Omron™HEM-7200, Dalian, China). Hypertension was confirmed if the participant met any of the following criteria: systolic BP ≥ 140 mmHg and/or diastolic BP ≥ 90 mmHg, and/or with self-reported physician-diagnosed hypertension, and/or on anti-hypertensive drugs [25]. Glycated haemoglobin (HbA1c) was examined using high-performance liquid chromatography and plasma glucose was measured with hexokinase method. Diabetes mellitus was defined as random plasma glucose ≥11.1 mmol/L, and/or fasting plasma glucose ≥7.0 mmol/L, and/or HbA1c ≥ 6.5%, and/or with self-reported diabetes mellitus diagnosed by a physician, and/or on glucose lowering drugs or insulin treatment [26]. We also extracted 11 self-reported physician diagnosed diseases from the baseline dataset, including dyslipidaemia, heart disease, stroke, chronic lung disease, asthma, liver disease, cancer, digestive disease, kidney disease, arthritis, and memory-related disease (including Alzheimer’s disease, Parkinson’s disease, and cerebral atrophy). Multimorbidity was defined as the presence of two or more of the above 13 chronic conditions in the same individual [27–29].

### Statistical analysis

To describe the baseline characteristics, mean ± standard deviation (SD) was used for continuous variables, and frequency (percentage) was applied for categorical variables. Analysis of variance (ANOVA) and Chi-square tests were conducted to compare differences of continuous variables and categorical variables across the four depression-obesity groups at baseline, respectively. Comparisons of baseline characteristics between the included and excluded individuals were further conducted with independent student t-test or Chi-square tests, where appropriate.

In the first analysis, logistic regression models were established to evaluate the associations between baseline depression-obesity status and incident functional disability. Model 1 was a crude model. Model 2 was adjusted for baseline age, gender, education level, current marital status, area of residence, smoking and drinking status. Model 3 was additionally adjusted for multimorbidity status, as previous research has indicated its detrimental impact on functional disability [30]. Odds ratios (OR) and 95% confidence intervals (CI) were presented.

In the second analysis regarding the transition of depression and obesity status, a Sankey diagram was depicted to directly visualize the number of participants transitioning across different groups from baseline to follow-up. To determine the association between changes in depression-obesity status and the risk of developing functional disability, participants were divided into three categories: (1) stability (3 groups); (2) progression (2 groups); and (3) regression (2 groups). The stability category was further divided into 3 groups, including participants with neither condition, single condition (depression alone or obesity alone), and comorbidity throughout the baseline and follow-up surveys. Participants with depression alone or obesity alone were combined together in the analysis as the risk of functional disability were similar in these two groups according to our results. Additionally, the number of individuals changed from depression alone to obesity alone or vice versa was relatively small, while combination of these individuals into a single group enabled us to perform analyses with sufficient statistical power. The progression category included 2 groups, i.e., participants progressed from neither condition at baseline to single condition at follow-up, and participants progressed from 0 or 1 condition at baseline to comorbidity at follow-up. The regression category also included 2 groups, one group with participants regressed from comorbidity to single condition, and the other group with participants regressed from comorbidity or single condition at baseline to neither condition at follow-up. Three logistic regression models with the same covariates adjusted in the first analysis were constructed, with the group of neither condition throughout the baseline and follow-up surveys treated as the reference.

All analyses were conducted with Stata/SE 16.0 (Stata-Corp, College Station, TX). Statistical significance was determined as two-tailed *P* < 0.05.

## Results

Of the 5507 participants enrolled in the first analysis, the mean age was 60.6 ± 9.4 years and 62.4% were females (Table 1). The numbers of participants with neither condition, depression alone, obesity alone, and comorbid depression and obesity were 2384 (43.3%), 2390 (43.4%), 447 (8.1%), and 286 (5.2%), respectively. Compared to participants with neither condition, individuals with comorbid depression and obesity were younger, more likely to be females, had higher levels of BP and glucose profiles, as well as higher prevalence of multimorbidity. We also observed statistically significant difference between the included and excluded individuals for most of the baseline characteristics (Supplementary Table 1).

**Table 1:** Comparison of characteristics across different depression and obesity groups at baseline

	All participants	Neither condition	Depression alone	Obesity alone	Comorbidity^a^	***P*** value
**N**	5507	2384 (43.3%)	2390 (43.4%)	447 (8.1%)	286 (5.2%)	
**Socio-demographic and lifestyle factors**						
Mean age (years), mean (SD)	60.6 ± 9.4	61.2 ± 9.6	60.9 ± 9.3	58.0 ± 8.8	57.9 ± 8.2	< 0.001
Gender, n (%)						
Male	2072 (37.6%)	1052 (44.1%)	831 (34.8%)	130 (29.1%)	59 (20.6%)	< 0.001
Female	3435 (62.4%)	1332 (55.9%)	1559 (65.2%)	317 (70.9%)	227 (79.4%)	
Education level, n (%)						
Illiterate/no formal education	3002 (54.5%)	1203 (50.5%)	1438 (60.2%)	197 (44.1%)	164 (57.3%)	< 0.001
Primary school	1248 (22.7%)	558 (23.4%)	532 (22.3%)	99 (22.1%)	59 (20.6%)	
Middle school or above	1257 (22.8%)	623 (26.1%)	420 (17.6%)	151 (33.8%)	63 (22.0%)	
Current marital status, n (%)						
Married or cohabitated	4679 (85.0%)	2074 (87.0%)	1948 (81.5%)	411 (91.9%)	246 (86.0%)	< 0.001
Not married^b^	828 (15.0%)	310 (13.0%)	442 (18.5%)	36 (8.1%)	40 (14.0%)	
Area of residence, n (%)						
Urban	1762 (32.0%)	813 (34.1%)	635 (26.6%)	209 (46.8%)	105 (36.7%)	< 0.001
Rural	3745 (68.0%)	1571 (65.9%)	1755 (73.4%)	238 (53.2%)	181 (63.3%)	
Ever smoker, n (%)	1885 (34.2%)	935 (39.2%)	787 (32.9%)	106 (23.7%)	57 (19.9%)	< 0.001
Ever drinker, n (%)	1914 (34.8%)	868 (36.4%)	852 (35.7%)	130 (29.1%)	64 (22.4%)	< 0.001
**Clinical / biochemical measures, mean (SD)**						
BMI (kg/m^2^)	23.6 ± 4.1	22.8 ± 2.9	22.3 ± 2.9	30.6 ± 3.3	30.8 ± 3.6	< 0.001
Systolic BP (mmHg)	131.3 ± 21.9	130.9 ± 21.8	129.6 ± 22.0	138.5 ± 21.1	136.6 ± 20.3	< 0.001
Diastolic BP (mmHg)	75.6 ± 12.1	75.4 ± 11.9	74.2 ± 12.0	80.9 ± 11.8	80.4 ± 11.4	< 0.001
HbA1c (%)	5.3 ± 0.8	5.2 ± 0.8	5.3 ± 0.8	5.4 ± 0.8	5.6 ± 1.0	< 0.001
Plasma glucose (mmol/L)	6.1 ± 1.9	6.1 ± 1.8	6.1 ± 2.0	6.4 ± 2.0	6.5 ± 2.1	< 0.001
Multimorbidity, n (%)						
No	2380 (44.9%)	1227 (53.2%)	924 (40.3%)	150 (35.4%)	79 (28.4%)	< 0.001
Yes	2925 (55.1%)	1081 (46.8%)	1371 (59.7%)	274 (64.6%)	199 (71.6%)	

*Abbreviation*: *BMI* Body mass index, *BP* Blood pressure, *HbAlc* Glycated haemoglobin, *SD* Standard deviation

^a^Comorbidity was defined as the co-existence of depression and obesity

^b^Not married included separated, divorced, widowed, and never married

After 4 years of follow-up, 510 (9.3%) participants developed functional disability. The incidence of functional disability was the highest among participants with comorbid depression and obesity (15.7%), followed by those with depression alone (10.6%) and obesity alone (9.6%), and was the lowest among individuals with neither condition (7.1%). Similar patterns were observed for the incidence of functional disability in the dressing, bathing, eating, transferring, and toileting subscales, except for the incidence of continence, which was comparable across different depression-obesity groups (Supplementary Figure 1). Table 2 shows the association between baseline depression-obesity status and incident functional disability, as well as each ADL item. In the unadjusted model, compared to participants with neither depression nor obesity, those with depression alone had increased risk of functional disability (OR = 1.55, 95% CI: 1.27–1.90), while the risk estimate was not significant for obese participants without depression (OR = 1.39, 95% CI: 0.98–1.98). Additionally, comorbid depression and obesity was associated with the highest risk of functional disability with an OR of 2.45 (95% CI: 2.72–3.49). In the adjusted model 2 and 3, we observed similar findings, except that participants with obesity alone also showed significantly increased risk of functional disability. We further found that compared to participants with neither condition, or with either condition alone at baseline, those with comorbid depression and obesity had greater risk of disability in dressing, bathing, eating, transferring, and toileting, but not in continence.

**Table 2:** Association between baseline depression-obesity status and the risk of functional disability

	Neither condition	Depression alone		Obesity alone		Comorbidity^d^	
		OR (95% CI)	***P*** value	OR (95% CI)	***P*** value	OR (95% CI)	***P*** value
**Functional disability**^e^							
Model 1^a^	1.00 (ref)	1.55 (1.27, 1.90)	< 0.001	1.39 (0.98, 1.98)	0.063	2.45 (1.72, 3.49)	< 0.001
Model 2^b^	1.00 (ref)	1.58 (1.28, 1.95)	< 0.001	1.72 (1.20, 2.47)	0.003	3.06 (2.12, 4.43)	< 0.001
Model 3^c^	1.00 (ref)	1.52 (1.23, 1.88)	< 0.001	1.54 (1.06, 2.26)	0.024	2.84 (1.95, 4.15)	< 0.001
**Disability in dressing**							
Model 1	1.00 (ref)	1.35 (0.92, 1.99)	0.124	1.76 (0.98, 3.19)	0.060	3.01 (1.68, 5.39)	< 0.001
Model 2	1.00 (ref)	1.36 (0.92, 2.02)	0.120	2.22 (1.21, 4.06)	0.010	3.76 (2.06, 6.84)	< 0.001
Model 3	1.00 (ref)	1.29 (0.87, 1.92)	0.210	2.14 (1.16, 3.94)	0.014	3.29 (1.77, 6.12)	< 0.001
**Disability in bathing**							
Model 1	1.00 (ref)	1.50 (1.15, 1.97)	0.003	1.41 (0.89, 2.24)	0.142	2.81 (1.82, 4.35)	< 0.001
Model 2	1.00 (ref)	1.59 (1.20, 2.10)	0.001	1.83 (1.13, 2.94)	0.013	3.82 (2.42, 6.04)	< 0.001
Model 3	1.00 (ref)	1.50 (1.12, 1.99)	0.006	1.72 (1.06, 2.80)	0.029	3.41 (2.13, 5.48)	< 0.001
**Disability in eating**							
Model 1	1.00 (ref)	1.47 (0.76, 2.84)	0.254	2.15 (0.83, 5.57)	0.115	3.96 (1.60, 9.80)	0.003
Model 2	1.00 (ref)	1.55 (0.80, 3.03)	0.196	2.77 (1.05, 7.35)	0.040	5.89 (2.30, 15.12)	< 0.001
Model 3	1.00 (ref)	1.35 (0.68, 2.68)	0.396	2.49 (0.93, 6.65)	0.069	5.12 (1.97, 13.32)	0.001
**Disability in transferring**							
Model 1	1.00 (ref)	1.63 (0.98, 2.72)	0.061	1.79 (0.80, 4.01)	0.157	3.56 (1.69, 7.53)	0.001
Model 2	1.00 (ref)	1.68 (1.00, 2.83)	0.051	2.52 (1.10, 5.79)	0.029	5.19 (2.38, 11.34)	< 0.001
Model 3	1.00 (ref)	1.55 (0.91, 2.64)	0.105	2.38 (1.03, 5.50)	0.042	4.38 (1.93, 9.92)	< 0.001
**Disability in toileting**							
Model 1	1.00 (ref)	1.25 (0.93, 1.69)	0.137	1.76 (1.11, 2.76)	0.015	2.60 (1.62, 4.18)	< 0.001
Model 2	1.00 (ref)	1.24 (0.91, 1.68)	0.172	2.33 (1.45, 3.73)	< 0.001	3.46 (2.11, 5.67)	< 0.001
Model 3	1.00 (ref)	1.19 (0.87, 1.63)	0.288	2.02 (1.23, 3.33)	0.006	3.26 (1.96, 5.43)	< 0.001
**Disability in continence**							
Model 1	1.00 (ref)	1.43 (0.95, 2.15)	0.086	1.34 (0.67, 2.70)	0.413	1.47 (0.65, 3.31)	0.354
Model 2	1.00 (ref)	1.51 (0.99, 2.28)	0.054	1.77 (0.86, 3.62)	0.119	2.06 (0.90, 4.73)	0.087
Model 3	1.00 (ref)	1.48 (0.97, 2.26)	0.068	1.78 (0.87, 3.67)	0.115	2.06 (0.89, 4.76)	0.089

*Note:*^a^ Model 1, unadjusted. ^b^ Model 2, adjusted for baseline age, gender, area of residence, marital status, education level, smoking and drinking status. ^c^ Model 3, additionally adjusted for baseline multimorbidity status. *Abbreviation*: *OR* Odds ratios, *CI* Confidence interval

^d^Comorbidity was defined as the co-existence of depression and obesity

^e^Functional disability was defined as any limitation in dressing, bathing, eating, transferring, toileting, or continence

**Fig. 2:**
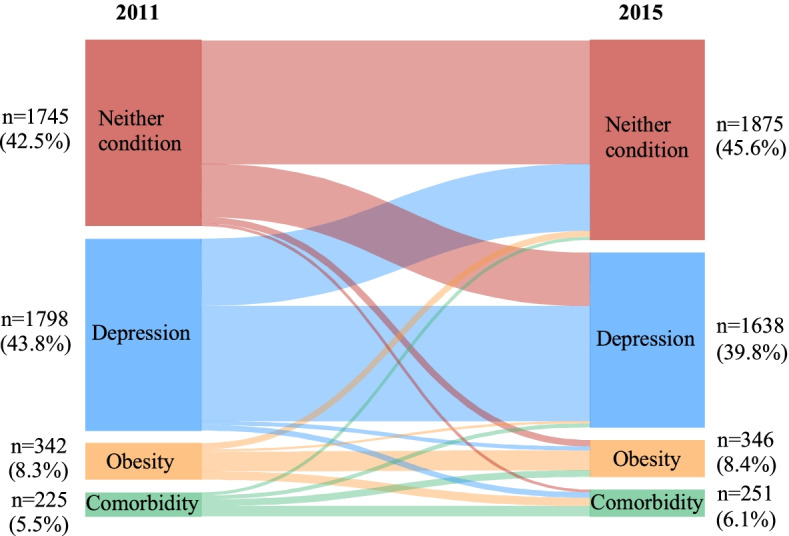
Transition of depression-obesity status between baseline and follow-up. The width of the connecting edges is proportional to the number of individuals transitioning between groups at baseline (left side) and follow-up (right side). Neither condition: No depression and no obesity; Comorbidity: with both depression and obesity

Of the 4110 participants included in analysing the dynamic change of depression-obesity status from baseline to follow-up, 2524 individuals maintained the same depression-obesity status, 814 participants converted to a more favourable group, and 714 transferred to a worse condition status (shown in Fig. 2). We further evaluated the association between transition of depression-obesity status over time with the risk of functional disability (Table 3). Compared to individuals maintained no depression and no obesity at both baseline and follow-up, those with comorbid depression and obesity throughout both surveys were at the greatest risk of functional disability in the fully adjusted model (OR = 4.06, 95% CI: 2.11–7.80), followed by the group that progressed from neither or single condition at baseline to comorbidity at follow-up (OR = 3.10, 95% CI: 1.73–5.55), and the group regressed from comorbidity to single condition (OR = 2.83, 95% CI: 1.43–5.61). Furthermore, participants with single condition throughout the two surveys (OR = 2.10, 95% CI: 1.49–2.96) or progressed from neither condition to single condition (OR = 1.92, 95% CI: 1.27–2.91) had approximately twice increased risk of functional disability compared to the reference group. In contrast, we did not find significantly increased risk of functional disability among participants who regressed from comorbidity or single condition at baseline to neither depression nor obesity at follow-up (OR = 0.94, 95% CI: 0.60–1.48).

**Table 3:** Association between transition in depression-obesity status and the risk of functional disability

Transition of depression-obesity status	Model 1^a^		Model 2^b^		Model 3^c^	
	OR (95% CI)	***P*** value	OR (95% CI)	***P*** value	OR (95% CI)	***P*** value
**Stability**						
Neither throughout	1.00 (ref)	–	1.00 (ref)	–	1.00 (ref)	–
Single condition throughout	2.04 (1.47, 2.83)	< 0.001	2.15 (1.53, 3.02)	< 0.001	2.10 (1.49, 2.96)	< 0.001
Comorbidity throughout	3.39 (1.81, 6.35)	< 0.001	4.25 (2.22, 8.11)	< 0.001	4.06 (2.11, 7.80)	< 0.001
**Progression**						
Neither to single condition	1.80 (1.20, 2.70)	0.005	1.89 (1.25, 2.85)	0.003	1.92 (1.27, 2.91)	0.002
Neither/single condition to comorbidity	2.66 (1.52, 4.66)	0.001	3.09 (1.73, 5.51)	< 0.001	3.10 (1.73, 5.55)	< 0.001
**Regression**						
Comorbidity to single condition	2.77 (1.43, 5.37)	0.003	2.96 (1.50, 5.82)	0.002	2.83 (1.43, 5.61)	0.003
Comorbidity/single condition to neither	1.00 (0.65, 1.55)	0.989	0.99 (0.63, 1.54)	0.958	0.94 (0.60, 1.48)	0.801

*Note:*^a^ Model 1, unadjusted. ^b^ Model 2, adjusted for baseline age, gender, area of residence, marital status, education level, smoking and drinking status. ^c^ Model 3, additionally adjusted for baseline multimorbidity status

*Abbreviation*: *OR* Odds ratios, *CI* Confidence interval

## Discussion

This study revealed evidence that individuals with comorbid depression and obesity at baseline had the highest risk of developing functional disability, compared to those with neither condition, or with either condition alone. Our data further suggested that participants with stable comorbidity or progression to comorbidity had the highest risks of incident functional disability, whereas such risk estimates were relatively lower in those with single condition at follow-up. The risk was attenuated to null in individuals regressed to no depression and no obesity during follow-up.

The independent associations of depression and obesity with functional disability was in line with several previous studies [6–8]. We further demonstrated that the risk of functional disability was the highest in participants suffering from both depression and obesity. This was consistent with previous findings showing that comorbid depression and obesity had a greater negative impact on health and health-related quality of life than those with neither condition, or with either condition alone [12, 31–33]. The mechanisms underlying the greater detrimental impact of comorbid depression and obesity on functional disability have not been fully understood. Previous evidence has shown that depression was associated with dysregulation of the prefrontal cortex circuitry, the brain region that is responsible for executive function [34]. Executive dysfunction could consequently elevate the risk of impairment in functional ability [35]. In terms of the association between obesity and functional disability, excessive body weight might cause musculoskeletal injuries by increasing the loading of weight-bearing joints mechanically [36], subsequently leading to hip and knee osteoarthritis and ultimately the functional disability [37–39]. Therefore, depression and obesity could independently increase the risk of functional disability, and such deterioration might be exaggerated when individuals had depression and obesity simultaneously.

In regard to the associations between different depression-obesity groups and the risk of each ADL item. We found that comorbid depression and obesity was significantly associated with increased risk of disability in dressing, bathing, eating, transferring, and toileting, but not in continence. The exact mechanism underlying this finding was unclear. One possible explanation might be related to the self-stigma of being incontinent. Therefore, disability in continence was more likely to be underreported compared to other disabilities, which might lead to an underestimation of the association [40, 41]. Another possible explanation for this discrepancy might be the residual confounding. Since some common risk factors for incontinence (e.g., urinary infection and gastrointestinal disorders) were not included in regression models, the impact of comorbid depression and obesity on disability in continence might have been attenuated. Nevertheless, further studies with larger sample size are needed to confirm the conclusion.

The increased risk of functional disability caused by comorbid depression and obesity might be due to several shared pathways. First, depression and obesity have shown synergistic effect on the risk of chronic diseases [32], which were well-established risk factors of functional disability [42]. Second, individuals with comorbid depression and obesity had markedly higher level of inflammation than those with either condition alone [43]. The elevated levels of inflammatory cytokines, such as interleukin-6, C-reactive protein, and fibrinogen, could further result in poorer physical performance, greater functional limitations, and increased risk of later-life disability [44, 45]. In addition, inflammation could lead to endothelial dysfunction [46], a key component of atherosclerosis that contributes to the development of peripheral and cerebrovascular diseases [47], which could further induce physical dysfunction and disability [48, 49]. Third, individuals with comorbid depression and obesity usually had poor adherence to healthy lifestyle and were more likely to be physical inactive [50], which was associated with greater risk of functional disability in older adults [51]. Therefore, the greater detrimental effect of comorbid depression and obesity on functional disability observed in our study was plausible.

Our study further explored the impact of transition in depression-obesity status over time on the risk of functional disability. The results confirmed that progression to a less favourable condition status was accompanied with increases in the risk of developing functional disability. In contrast, the risk estimates of functional disability were reduced in participants with regression of depression-obesity status from baseline to follow-up. The findings were in line with previously published literatures showing that treatment of depression or weight loss could restore functional ability [52, 53]. Furthermore, a randomized controlled trial has demonstrated that compared to usual care, an integrated collaborative care intervention for individuals with comorbid depression and obesity could improve functional outcome measured by the Sheehan Disability Scale at 6 months, but not at 12 months [54]. Our research highlighted the possibility that interventions of psychotherapy or increasing exercise among middle-aged and older adults with comorbid depression and obesity might have beneficial effect on functional ability in the long term. However, further randomized controlled trials are needed to confirm the findings.

The strengths of this study include the longitudinal design, the relatively large study sample, the repeated measurements, and the validated questionnaires used to measure depression and functional disability. A further strength is that we also investigated how the transition of depression-obesity status affected the onset of functional disability, which provide important clinical implication for prevention and intervention of functional disability in middle-aged and older Chinese adults. However, some limitations merit discussion. First, a number of individuals were excluded from this study due to missing information or loss to follow-up. In addition, several baseline characteristics were statistically different between the included and excluded individuals, imposing concerns on the generalizability of our study. Nevertheless, results obtained from the imputed datasets also demonstrated increased risk of functional disability in those with comorbid depression and obesity (data not shown), indicating the robustness of our findings. Second, depression was ascertained by CESD-10 instead of the gold-standard clinical diagnoses, which might partially cause misclassification bias. However, CESD-10 has been demonstrated to be reliable and valid in epidemiology research among older adults in China [22]. Third, despite our efforts to control for several potential confounders, residual confounding might persist [52, 55]. However, we were unable to adjust variables such as physical activity and antidepressant drug use due to data unavailability. Last, the number of individuals with a transition from neither condition to comorbidity (*N* = 25) or vice versa (*N* = 29) was relatively small in our study, thus we were unable to explore their risk of developing functional disability. Future studies are needed to further confirm the findings.

## Conclusion

In conclusion, our study shows that comorbid depression and obesity could exert greater detrimental effects on the risk of functional disability among middle-aged and older adults in China. Importantly, our data suggest that depression and obesity status is transient, with the highest functional disability risk for those who remain comorbidity. The risk of functional disability was increased with deterioration in depression-obesity status and reduced with regression of depression and obesity. Apart from body weight monitoring, our findings highlight the need of screening depression among middle-aged and older adults, especially for obese adults, which may help identify the high-risk population of developing functional disability.

## Supplementary Information

**Additional file 1: Supplementary Table 1.** Comparison of baseline characteristics between included and excluded individuals. **Supplementary Figure 1.** Incidence of functional disability in 2015 in different groups according to baseline depression-obesity status.

## Data Availability

Data of this study are available on the website of the China Health and Retirement Longitudinal Study (CHARLS) at http://charls.pku.edu.cn/index/en.html. To access and use this survey data for research purpose, an approval should be obtained from the CHARLS team at Peking University.

## Abbreviations

ADL: Activity of daily living
ANOVA: Analysis of variance
BMI: Body mass index
BP: Blood pressure
CESD-10: 10-item Centre for Epidemiological Studies Depression Scale
CHARLS: China Health and Retirement Longitudinal Study
CI: Confidence interval
HbAlc: Glycated haemoglobin
OR: Odds ratio
PPS: Probability-proportional-to-size
SD: Standard deviation
