# Supplementary material for: Comorbid depression and obesity, and its transition on the risk of functional disability among middle-aged and older Chinese: a cohort study
Source: BMC Geriatr. 2022 Apr 3;22:275. doi: 10.1186/s12877-022-02972-1 (PMC8976974; doi:10.1186/s12877-022-02972-1)
Supplement: Supplementary file 1 — Additional file 1: Supplementary Table 1. Comparison of baseline characteristics between included and excluded individuals. Supplementary Figure 1. Incidence of functional disability in 2015 in different groups according to baseline depression-obesity status. [file 12877_2022_2972_MOESM1_ESM.docx]

**Supplementary Table 1. Comparison of baseline characteristics between included and excluded individuals**

|  |  | **Included** | **Excluded** | ***P* value** |
| --- | --- | --- | --- | --- |
| **N** | | 4110 | 13598 |  |
| **Socio-demographic and lifestyle factors** | |  |  |  |
| Mean age (years), mean (SD) | | 59.76 (8.71) | 58.71 (10.62) | <0.001 |
|  | Missing | 0 | 11 |  |
| Gender, n (%) | |  |  | <0.001 |
|  | Male | 1556 (37.9%) | 6898 (50.7%) |  |
|  | Female | 2554 (62.1%) | 6697 (49.3%) |  |
|  | Missing | 0 | 3 |  |
| Education, n (%) | |  |  | <0.001 |
|  | Illiterate/no formal education | 2155 (52.4%) | 5788 (42.7%) |  |
|  | Primary school | 988 (24.0%) | 2824 (20.9%) |  |
|  | Middle school or above | 967 (23.5%) | 4931 (36.4%) |  |
|  | Missing | 0 | 55 |  |
| Current marital status, n (%) | |  |  | 0.524 |
|  | Married or cohabitated | 3573 (86.9%) | 11844 (87.3%) |  |
|  | Not married^1^ | 537 (13.1%) | 1721 (12.7%) |  |
|  | Missing | 0 | 33 |  |
| Area of residence, n (%) | |  |  | <0.001 |
|  | Urban | 1257 (30.6%) | 5913 (43.5%) |  |
|  | Rural | 2853 (69.4%) | 7684 (56.5%) |  |
|  | Missing | 0 | 1 |  |
| Ever smoker, n (%) | | 1432 (34.8%) | 4879 (38.1%) | <0.001 |
|  | Missing | 0 | 801 |  |
| Ever drinker, n (%) | | 1463 (35.6%) | 5747 (42.8%) | <0.001 |
|  | Missing | 2 | 163 |  |
| **Clinical / biochemical measures, mean (SD)** | | |  |  |
| BMI (kg/m^2^) | | 23.75 (4.01) | 23.35 (3.79) | <0.001 |
|  | Missing | 0 | 4113 |  |
| Systolic BP (mmHg) | | 130.39 (21.46) | 130.90 (21.77) | 0.214 |
|  | Missing | 20 | 3914 |  |
| Diastolic BP (mmHg) | | 75.47 (11.91) | 76.19 (12.36) | 0.002 |
|  | Missing | 20 | 3912 |  |
| HbA1c (%) | | 5.28 (0.80) | 5.25 (0.83) | 0.069 |
|  | Missing | 860 | 5142 |  |
| Plasma glucose (mmol/L) | | 6.13 (1.89) | 6.13 (2.14) | 0.866 |
|  | Missing | 895 | 5177 |  |
| Multimorbidity, n (%) | |  |  | <0.001 |
|  | No | 1809 (44.6%) | 7809 (59.1%) |  |
|  | Yes | 2243 (55.4%) | 5396 (40.9%) |  |
|  | Missing | 58 | 393 |  |

Abbreviation: BMI, Body mass index; BP, Blood pressure; HbAlc, Glycated haemoglobin; SD: Standard deviation.

^1^Not married included separated, divorced, widowed, and never married.


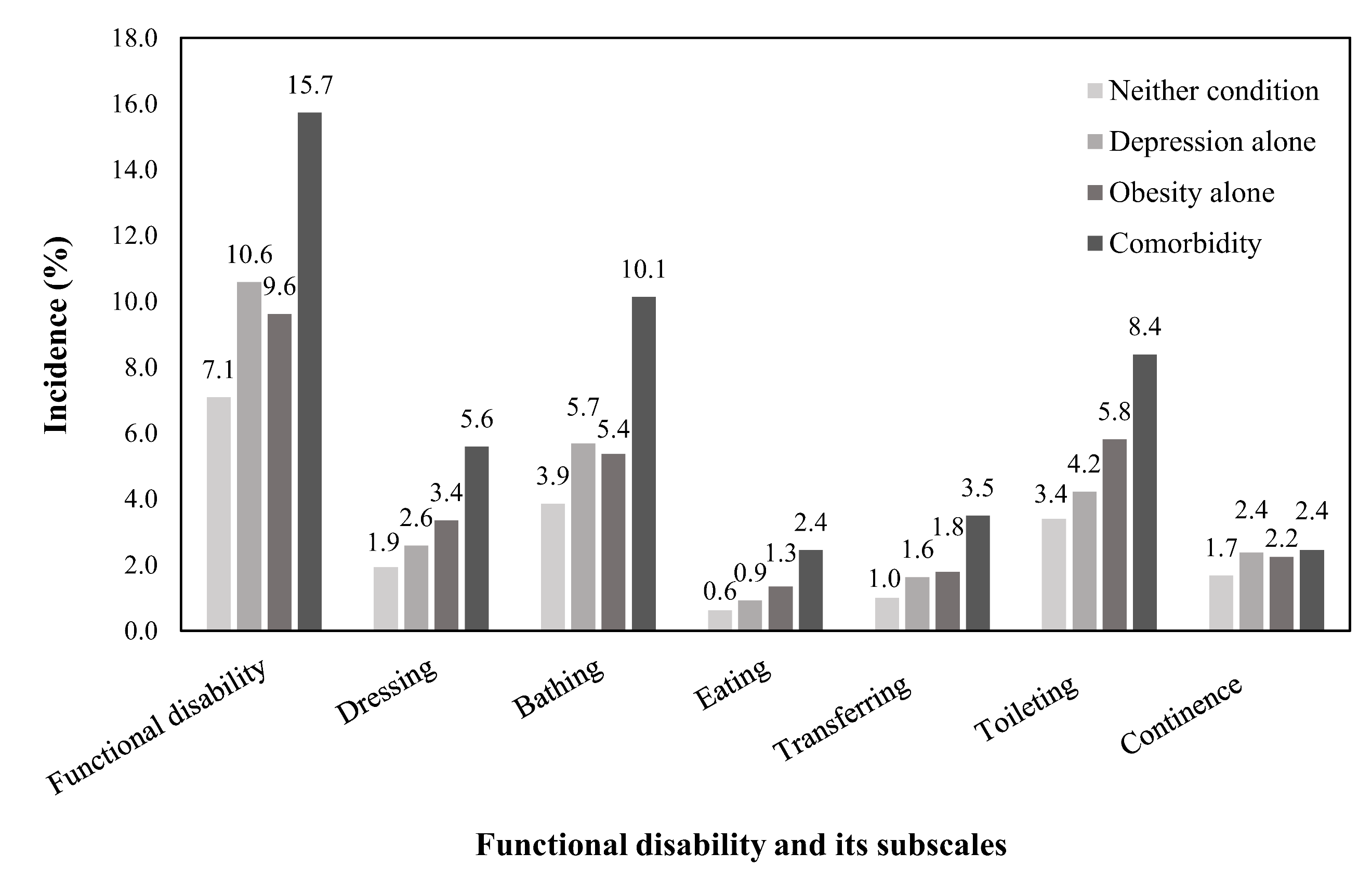


**Supplementary Figure 1. Incidence of functional disability in 2015 in different groups according to baseline depression-obesity status**

^1^Comorbidity was defined as the co-existence of depression and obesity.

^2^Functional disability was defined as any limitation in dressing, bathing, eating, transferring, toileting, or continence.
